# Comprehensive Overview of Gene Rearrangements in Childhood T-Cell Acute Lymphoblastic Leukaemia

**DOI:** 10.3390/ijms22020808

**Published:** 2021-01-15

**Authors:** Anna Mroczek, Joanna Zawitkowska, Jerzy Kowalczyk, Monika Lejman

**Affiliations:** 1 Department of Paediatric Haematology, Oncology and Transplantology, Medical University of Lublin, 20-093 Lublin, Poland; anna.mroczek94@wp.pl (A.M.); jzawitkowska1971@gmail.com (J.Z.); jerzy.kowalczyk@uszd.lublin.pl (J.K.); 2 Laboratory of Genetic Diagnostics, Medical University of Lublin, 20-093 Lublin, Poland

**Keywords:** T-ALL, genomic landscape, paediatrics

## Abstract

Acute lymphoblastic leukaemia (ALL) is a relevant form of childhood neoplasm, as it accounts for over 80% of all leukaemia cases. T-cell ALL constitutes a genetically heterogeneous cancer derived from T-lymphoid progenitors. The diagnosis of T-ALL is based on morphologic, immunophenotypic, cytogenetic, and molecular features, thus the results are used for patient stratification. Due to the expression of surface and intracellular antigens, several subtypes of T-ALL can be distinguished. Although the aetiology of T-ALL remains unclear, a wide spectrum of rearrangements and mutations affecting crucial signalling pathways has been described so far. Due to intensive chemotherapy regimens and supportive care, overall cure rates of more than 80% in paediatric T-ALL patients have been accomplished. However, improved knowledge of the mechanisms of relapse, drug resistance, and determination of risk factors are crucial for patients in the high-risk group. Even though some residual disease studies have allowed the optimization of therapy, the identification of novel diagnostic and prognostic markers is required to individualize therapy. The following review summarizes our current knowledge about genetic abnormalities in paediatric patients with T-ALL. As molecular biology techniques provide insights into the biology of cancer, our study focuses on new potential therapeutic targets and predictive factors which may improve the outcome of young patients with T-ALL.

## 1. Introduction

Acute lymphoblastic leukaemia (ALL) is the most frequent neoplasm in paediatric patients. Among ALL cases in children, ~15% represent diseases derived from T cells, and the remaining ~85% consist of B cell precursor (BCP)-ALL [1,2,3]. The ALL subtypes occur among adults with a frequency of ~25 and ~75%, respectively [1,3,4,5]. Due to the expression of surface and intracellular antigens, we distinguish several subtypes of T-ALL. The European Group for Immunologic Classification (EGIL) scale is used to classify T-ALL into subtypes. EGIL T-I corresponds to pro-T (characterized by cCD3+, CD7+); EGIL T-II is pre-T (cCD3+, CD7+, CD5/CD2+); EGIL T-III is cortical-T (cCD3+, Cd1a+, sCD3+/–); and EGIL T-IV corresponds to mature-T (cCD3+, sCD3+, CD1a-) and T-γ/δ, with observed frequencies of 5.1, 31.4, 38.1, 15.3, and 10.2% respectively [2,6,7,8]. However, this classification does not inform us about the prognostic value of individual groups.

The development of medicine and the modification of therapeutic protocols lead to increasingly improved results in ALL paediatric patients in terms of total remission and long-term survival. The 5-year survival rate for ALL patients under 20 years of age has improved significantly over time and is currently 90% [9]. However, there is still a group of patients who do not achieve such results [3,10]. According to reports, every fifth child suffering from T-ALL experiences a relapse or dies due to this disease [11,12,13]. The main cause of death of patients with ALL is relapse [11,14]. Relapse is a result of the proliferation of the most resistant cells that survive after previous treatment. Therefore, therapy for relapse is limited due to the observed drug resistance and shows poorer results compared to the treatment of the primary disease [15,16]. Moreover, marked intensification of therapy among young patients will not resolve the problem because of reduced tolerance to cytotoxic substances and severe side effects [3,10,11,12]. Therefore, alternative forms of therapy are required. It is necessary to optimize the treatment based on patient classification using molecular genetic markers and individual genetic profiles [2,12,17,18].

In recent years, a subgroup of early T-cell precursor (ETP)-ALL has been identified among patients, which is characterized by worse prognosis and chemoresistance in comparison to non-ETP-ALL [1]. ETP is derived from thymocytes, which are capable of differentiating into T cells and marrow cells, which proves their hematopoietic nature [19,20]. In paediatric T-ALL/acute lymphoblastic lymphoma (LBL), the ETP-ALL subgroup represents 11–13% cases, while in adults it constitutes 7.4%, but some studies indicate that ETP-ALL occurs in 15 and 10–30%, respectively [2,20,21,22,23]. Patients with ETP-ALL are significantly older at diagnosis, have a higher percentage of blasts in the marrow or peripheral blood, and circulating white blood cell (WBC) counts less than 50 × 109/L in comparison to patients with non-ETP-ALL [24]. Immunophenotypic screening criteria are used to diagnose ETP-ALL. ETP-ALL is characterized by the absence of both CD1a and CD8 (absent in more than 95% of leukemic cells), the absence or weak expression of CD5 (in ≤75% of total cells or 10 times less than in normal T cells), or the expression of at least one stem cell marker/stem cell: CD117, CD34, HLA-DR, CD13, CD33, CD11b, or CD65 (equal or more than 25% of total cells) [23]. In terms of immunophenotype, chromosomal aberrations, and genetic mutations, ETP-ALL is more similar to a myeloid malignancy, therefore there may be a need to separate this group from all T-ALL cases in order to apply another targeted treatment [25,26].

Despite immunophenotypic heterogeneity and interchangeability, T-ALL is associated with significant genetic heterogeneity. Leukemic transformation occurs through the accumulation of numerous genetic and epigenetic abnormalities [27]. They cause disorders of cell differentiation, apoptosis, activation of oncogenes, and inhibition of suppressors, as a result of excessive neoplastic cell proliferation. Chromosome aberrations were the first genetic changes observed in T-ALL patients, occurring in half of them [9]. Kowalczyk et al. in 1983 were the first to discover the genetic instability of chromosome 9p in patients diagnosed with T-ALL and suggested that it might correlate with poorer outcomes and reduced time of survival [28]. Intensified research in this field showed that of the genes located on the 9p21 deletion and promoter, hypermethylation of *CDKN2A* and *CDKN2B* genes has been observed in more than 70% of T-ALL patients. Through the use of advanced genome sequencing technology, mutations associated with the process of leukemogenesis by silencing cell cycle inhibitors and tumour suppressor genes were described. In both paediatric and adult patients with T-ALL, the most frequent gene mutation observed in 60% of cases was the *NOTCH1*, lying on the long arm of chromosome 9. The next most commonly reported gene alterations in ALL derived from T cell patients were *FBXW7* (8–30%), *PHF6* (20%), *PTEN* (20%), *WT1* (15%), *RUNX1* (10–20%), *LEF1* (10–15%), and *ETV6* (5–15%). As mentioned earlier, ETP-ALL shows some molecular and genetic similarity to myeloid malignancies. In ETP-ALL, mutations in *DNMT3A*, *ETV6*, *FLT3*, *GATA3*, *IDH1, IDH2*, *JAK3*, *NRAS*/*KRAS*, and *RUNX1* genes have been seen with greater frequency in comparison to non-ETP-ALL cases [17]. 

There is considerably more information about genetic abnormalities in acute lymphoblastic leukaemia from B cells compared to T-ALL. Due to its rare occurrence and high heterogeneity compared to BCP-ALL, the predictive factors and diagnostic markers of T-ALL are not well established. No genetic prognostic factor has been found for T-ALL so far. Therefore, T-ALL is the subject of intensive research among scientists [18]. In this review, we focus on a summary of knowledge about genetic alterations in T-ALL patients and their correlation with treatment response and prognosis, as well as the possibility to use them as points for prospective targeted therapies.

## 2. Genetic Profile of T-ALL

### 2.1. Transcription Factor

Chromosome aberrations were the first genetic changes observed in patients with T-ALL, and they occur in half of patients. It is supposed that chromosome aberrations initiate the process of carcinogenesis, but without accompanying copy number alterations (CNAs) they are not responsible for the formation of leukemic cells.

Oncogenic transcription factors can be divided into several groups: *TAL*, *LMO1*/2, *TLX1*/3, *LYL*, *HOXA*, *MEF2C*, *NKX1*, and *NKX2.* Aberrant expression of these factors is an element of T-ALL molecular pathogenesis (Table 1). Abnormal expression of specific transcription factors allow division of T-ALL patients into molecular subgroups [9,29,30,31]. Rearrangements involving the T lymphocyte receptor (T cell antigen receptor, TCR) are common changes in T-ALL and affect from 35% to almost half of patients [32,33]. With this in mind, T-ALL patients were divided into subgroups. The most common subtype emerging from mature late cortical thymocytes (CD4+ CD8+, CD3+) was characterized by *TAL1* with coexisting *LMO1*/2, affecting 30 to 37.5% of patients [29,33]. The second most frequent subtype (20–25%) had *HOXA-t*, *CALM-AF10*, and *KMT2A*-rearrangements (*KMT2A-R*). In the next subtype, with a frequency of 20–24%, abnormal expression of *TLX3* was observed. Additionally, it was observed that this group was characterized by more frequent loss of function of other genes due to mutations. Of T-ALL patients, 3–8% had aberrant expression of *TLX1.* The least numerous groups, representing 5.9 and 2.5%, were characterized by *NKX*-*1.-2* and *MEF2C*, respectively [29]. CD1a+, CD4+, CD8+ immunophenotype cells had complete ectopic expression of *TLX1*, *TLX3*, or *NKX-1.-2*, and ETP-ALL patients had abnormal expression of transcription factors *LMO2*/*LYL1* [33].

As many as one in three patients with diagnosed T-ALL have chromosomal translocations present in tumour cells t(10;14)(q24;q11) or through t(7;10)(q35;q24), leading to abnormal *HOX11 (TLX1)* activation. The high expression level of this gene was observed in 19.7% of patients, while low values were observed in 28.9%. Comparing the treatment results, both groups achieved similar outcomes. However, the analysis of samples of healthy patients did not show the presence of *HOX11* expression [34].

*KMT2A* are highly pathogenic leukemic drivers, revealed by the high incidence of *KMT2A*-rearangements in ALL in infants, with few cooperative mutations associated with leukaemia [1]. Rearrangement of the *KMT2A* (formerly *MLL*; 11q23) gene has been observed more frequently in paediatric patients with T-ALL than BCP-ALL, with a frequency of 4–8% [35,36]. Histone-lysine N-methyltransferase 2A is responsible for controlling the genes involved in the haematopoiesis process. More than 135 partner genes that merge with *KMT2A* have been described in recent papers. In a study conducted by Peterson et al., *KMT2A* was most often accompanied by partner genes such as *MLLT1* (19p13.3), *AFDN* (formerly *AF6*-6q27), *ELL* (19p13.11), and *MLLT10* (10p12.31). These were observed in respectively 33, 30, 7, and 4% of T-ALL patients aged 1–20 years. Interestingly, none of the patients had *KMT2A* fusions with *AFF1* (4q21) and *MLLT3* (9p21.3) genes, which is more characteristic of AML and BCP-ALL. It is worth noting that mergers with *KMT2A* tend to have a poor prognosis, but of all gene partners, *MLLT1* has a better prognosis in T-ALL/LBL [36].

Mansur et al. suggested that *KMT2A* rearrangements and *NOTCH1* mutations may be the first changes responsible for the process of T-ALL in infants. Fifteen patients under 2 years of age with T-ALL were studied. It was observed that the most common alterations in this group were *NOTCH1* mutations and *KMT2A*-R, which often involved partner genes: *MLLT1* and *AFF1* [37]. In research carried out by Matlawska-Wasowska, 12% of patients had *KMT2A*-related rearrangements. The most common partner genes were *MLLT4* (every third patient), del3′*KMT2A* (25%), *MLLT1* (25%), and *MLLT6-17q12* (8.3%) [38]. ALL patients with *KMT2A-R* are at high risk and represent a major therapeutic challenge. Only in less than 60% of cases is long-term survival achieved. Infants are a special age group, characterized by a severe course and increased risk of relapse [39]. To summarize, *KMT2A*-R occurs in paediatric patients with T-ALL at a frequency of 4–12%, often involving the *MLLT4* and *MLLT1* partner gene. *KMT2A*-R could play an important role in leukaemia development and may be related to poor prognosis [40].

Abnormalities in the protooncogene group of *MYB* (6q23) and *MYC* (8q24) genes were observed in patients with paediatric T-ALL. In a study conducted by Liu, alteration of *MYB* was found in 18.6% of cases. Of these, 12.5% were amplified, 4.92% mutated, and 4.17% rearranged. *TCRB* and *SLC12A9* are the partner genes that are merged with the 5′ region of *MYB*, whereas the *MYB*-R 3′ region in involved *PLAGL1*, *BDP1*, and *CHMP1A* partner genes [41]. Lejman et al. observed the coexistence of CNAs in *MYB* and *AIH1* (2q34) in 12.94% of paediatric patients with T-ALL, but these lesions were not observed separately [42]. Thakral et al. conducted research on 27 childhood and adult T-ALL patients and monitored *MYB* disorders with a similar frequency of 11% [43]. Translocation between chromosomes 6 and 7 was another way to activate the above-described gene [41]. *MYC* gene translocation was described in 6.1% of patients. This disorder occurred at a similar frequency in children and adults with T-ALL. In T-ALL, *MYC* translocations can be caused by chromosome 6 and 7 trisomy [44]. Excessive expression of *MYC* may also be due to gain-of-function *NOTCH1* mutations [45]. It is worth mentioning that changes in the PI3K/AKT path often leads to increased expression of *MYC* [46]. In patients with abnormal MYC, some genetic abnormalities were found with higher frequency than in other patients. *CDKN2A*/B deletions occurred in three-quarters of patients and *PTEN* deletion or mutation was observed in more than half. Studies on mouse models have shown that elevated expression of C-MYC closely correlates with T-ALL development, and its inhibition prevents T-ALL [44].

Mutations in the *RUNX1* (21q22.12) gene have been identified in 12.7% of patients. The role of this gene is assigned in early haematopoiesis. *RUNX1* abnormalities are dominant in patients with early T-ALL. Shorter overall survival (OS) was observed in patients in whom *RUNX1* mutations were accompanied by *CDKN2A* hypermethylation; however, due to too few patients, this result was not statistically significant. On the other hand, the coexistence of *RUNX1* mutation with mutations in *GATA3* and *SH2B3* genes led to a worse result in patients [17]. 

The *BCL11B* (14q32.2) gene is a regulator of T cell line differentiation and takes part in the conversion of cell precursors to mature T cells. 14q32.2of the *BCL11B* gene occurred in 2% of examined children. Moreover, this mutation was reported in as many as 16% of patients with *TLX1* overexpression [47]. In a study evaluated by Gutierrez et al., *BCL11B* gene mutation and deletion were found in 9% [48]. Its role in T-ALL development has not been defined yet [47].

The *ETV6* (12q13.2) gene takes part in haematopoiesis by influencing cell proliferation and differentiation. Deletion of *ETV6* was observed in 2 out of 36 (5.6%) paediatric patients with T-ALL [49]. The loss of heterozygosity of this gene was observed with a frequency of 6% in patients with T-ALL [50]. *ETV6/RUNX1* t(12;21)(p13;q22), which often co-occurs with deletion of the *ETV6* gene, is a typical rearrangement in childhood BCP-ALL, while in T-ALL, fusion-related signalling pathway or transcriptional regulation (*ETV6*/*ABL1* and *ETV6*/*CTNNB1*) was observed [41].

*GATA3* (10p14) belongs to the family of transcription factors and is involved in haematopoiesis. *GATA3* expression is key in transforming common lymphocyte progenitors into T lymphocytes and in differentiating Th2 effector cells [51]. Inactivating mutations of this gene have been reported in 9% of paediatric patients with ETP-ALL, representing about 10–15% of all T-ALL cases [52]. Analysis suggested that the signal transducer and activator of transcription (*STAT*)/Janus kinase (*JAK*) pathway activation in the *GATA3* regulatory network is a reliable mechanism for combining high expression of *GATA3* with ALL and therapy response. In a study conducted on peripheral T-cell lymphoma, *GATA3* overexpression was linked with poor OS [53].

*LEF1* (4q25) is a nuclear protein that is expressed in pre-B and -T cells and binds to a functionally important site in the T cell receptor alpha (TCRA) enhancer, and confers maximal enhancer activity [54]. In order to determine the significance of *LEF1* in the pathogenesis of T-ALL in children, Gutierrez et al. conducted a study involving 47 paediatric patients with diagnosed T-ALL. A biallelic or monoallelic deletion of tumour suppressor gene *LEF1* was noticed in 11% of them. Moreover, nonsynonymous sequence alterations of *LEF1* were detected with a frequency of 7% [55]. In another study, this CNA occurred in 10–24.5% of children and adult T-ALL cases [17]. Thakral et al. reported LEF1 alterations in 7.4% (*n* = 2) T-ALL patients [43]. Additionally, *LEF1* was most commonly observed in the *NKX2-1* T-ALL group [41]. Patients with *LEF1* inactivation were characterized by young age, thymocyte immunophenotype (CD1B+, CD1E+, CD8+, CD34-), increased expression of *MYC* and activation mutations of *NOTCH1*, *PI3K*, and *AKT*, *PTEN* inactivation mutations, and total inactivation of INK4A/ARF [55]. Opinions are divided on the role of *LEF1* inactivation in T-ALL. Montaño demonstrated that *LEF1* inactivation correlates with better OS in children and better response to salvage treatment [56]. In contrast, in a study conducted by Yeh et al., worse OS and a higher risk of relapse in *LEF1* deletion group of patients were observed [57].

*WT1* (11p13) gene encodes a zinc finger DNA-binding protein that acts as a transcriptional activator or repressor depending on the cellular or chromosomal context. *WT1* occurred in 13.2% of children with diagnosed T-ALL, 84% of which was heterozygous. The mutation most often concerned the C-terminal zinc finger fragment [58]. Liu et al. observed *WT1* in 9.1% of paediatric T-ALL patients [41]. A relationship between the occurrence of *WT1* mutation and abnormal expression of specific transcription factors *TLX1 (HOX11)*, *TLX3 (HOX11L2)*, and *HOXA9* was observed. This study excluded the association of *WT1* mutation with poor prognosis of patients. No differences in treatment response were observed. Five-year OS was similar in both groups: 60 ± 21% for the group with *WT1* mutation and 70 ± 8% for the group without [58].

*NF1* (17q11.2) gene encodes neurofibromin, a cytoplasmic protein that is predominantly expressed in leukocytes, among other cells. *NF1* regulates several intracellular processes, including the RAS-cyclic AMP pathway, the MAP kinase cascade, adenylyl cyclase, and cytoskeletal assembly [59]. Thakral et al., in a study involving a small cohort of patients, observed deletion of *NF1* and *SUZ12* (17q11.2) at 7.4% each. The loss of this gene function was related to poor prognosis and failure of treatment induction [43]. Yeh et al. did not report any case of *NF1* or *SUZ12* deletion [57]. *SUZ12* is a component of polycomb group complexes (PRCs) 2, 3, and 4, which are critical for proper embryonic development [60].

*IKAROS* family members are involved in the process of haematopoiesis by regulating lymphocyte differentiation [61]. The loss of function of the *IKZF1* (7p12.2) gene as a result of deletion or mutation can lead to the development of T-ALL, because this gene encodes the IKAROS protein, which acts as a suppressor [62]. In a study carried out on 27 paediatric and adult T-ALL patients, Thakral et al. recorded a loss of *IKZF1* at 7.4% [43]. Deletion and mutation of *IKZF1* were observed in 2.8 and 2% of T-ALL cases, respectively [49,63]. Krzanowski et al. conducted a study examining *IKZF1* gene deletion in 24 children with T-ALL and 66 with BCP-ALL. He observed that patients with T-ALL were characterized by a lower incidence of *RB1* deletion compared to BCP-ALL (10.53 and 40.63%, respectively) [64]. It was suggested that the loss of *IKZF1* is related to poor outcome, especially in BCP-ALL patients [41]. The *IKZF2* gene (2q34) belongs to the IKAROS family and encodes the synthesis of zinc finger protein Helios, which is necessary for proper lymphocyte differentiation and which is assigned probable importance as a cancer development suppressor. Interestingly, an *IKZF2* gene microdeletion was observed in 3.4% T-ALL childhood cases [63].

The *PAX5* (9p13.2) gene is responsible for B-cell-specific protein activator factor (BSAP) transcription. Its role has been described mainly in the pathogenesis of BCP-ALL. However, recent studies suggest that the loss of its function may also occur in T-ALL. *PAX5* gene abnormalities occurred in 14% of T-ALL children [42]. Olsson and Thakral obtained similar results, with alterations in *PAX5* found in approximately 11% of childhood T-ALL cases. Both studies were conducted on a limited number of T-ALL patients [43,49]. The significance of loss of *PAX5* function in paediatric T-ALL remains unknown.

### 2.2. Signalling Pathway Regulators

#### 2.2.1. NOTCH1 Pathway

An often observed phenomenon in T-ALL patients is a mutation in *NOTCH1* (9q34.3) and *FBXW7* (4q31.3) genes. It is estimated that mutations in the *NOTCH1* gene are the most common genetic abnormality observed in T-ALL (Table 2). Despite such frequent occurrences, it turned out to be a positive prognostic factor. The *NOTCH1* gene is responsible for correct T cell differentiation. Mutations of this gene were observed in more than half of paediatric patients with T-ALL. The *NOTCH1* gene is also activated by chromosomal rearrangement–translation (7;9)(q34:q34.3), which leads to the active form of *NOTCH1* [68].

The *FBXW7* gene is located on chromosome 4, and its inactivating mutation plays a role in oncogenic *NOTCH1* path activation. The *NOTCH1* gene also influences the degradation of MYC and cyclin E. Its mutation is observed with a frequency from about 10 to even 20% of T-ALL cases, depending on the study.

A study conducted by Liu et al. showed a correlation between mutations in the *NOTCH1* and *FBXW7* genes and the prognosis of T-ALL patients. The activating mutation in *NOTCH1/FBXW7* genes was observed in 60.60%, whereas *NOTCH1* mutation affected 51.11% of patients, and for *FBXW7* gene it was 22.38% [68]. In 2017, a study on 264 T-ALL children, mutations in *NOTCH1* and *FBXW7* genes were found in 74.6 and 23.9%, respectively [41]. Patients with mutations in the *NOTCH1* gene (with or without *FBXW7* commutation) were characterized by longer 5-year OS and event-free survival (EFS) [68].

Patient samples were compared at the beginning of T-ALL disease and the moment of relapse. Mutation in *NOTCH1* was observed with similar frequency in patients at the time of diagnosis as during relapse, in 66.7 and 72.7%, respectively. However, much more often at the moment of primary diagnosis, mutations concerned *FBXW7* (20.0%), *DNM2*-19p13.2 (20.0%), and *PHF6* (20.0%) genes. In contrast, *NT5C2* 10q24.32-q24.33 gene mutation (27.3%) was observed among patients who relapsed. This mutation showed a link to the risk of relapse in the study [69].

The mutation of *NOTCH1* in two out of three patients concerned the heterodimerization domain (HD), and in 31.4% the -Pro-Gln-Ser-Thr- (PEST) domain. Moreover, almost 30% of patients had many *NOTCH1* mutations [41]. *NOTCH1* mutations involving the C-terminal sequence were observed (PEST). These were much more common in patients who had relapsed (58.3%) than in those who had not relapsed (16.7%) [69].

A study that included γ-secretase inhibitors (GSI) in the treatment was conducted, but it showed high toxicity and did not yield the expected results [70]. However, it was suggested that the administration of an antimalarial drug chloroquine (which also has an anti-*NOTCH1* effect, but with a different mechanism) would cause synergy of action and increased sensitivity to GSI and improved GSI therapeutic effects [71].

#### 2.2.2. PI3-AKT-mTOR Pathway

*PTEN* (10q23.31) is a tumour suppressor gene whose mutations are observed in numerous cancers [72]. Alterations in the *PTEN* gene were observed in 11–27% of paediatric patients with T-ALL [73]. The main causes of *PTEN* gene inactivation are monoallelic point mutations, gene removal, or microdeletions [73]. This gene encodes a protein that is the most important negative regulator of the *PI3/AKT/mTOR* pathway. The loss of *PTEN* gene function due to mutations and deletions leads to inhibition transformation of dephosphorylation phosphatidylinositol (3,4,5) P3 (PIP3) to phosphatidylinositol (4,5) P2 (PIP2). This results in over-activation of the *PI3/AKT/mTOR* path caused by excess PIP3 and increased AKT1 (serine-threonine protein kinase) activity. *PI3/AKT/mTOR* has a regulatory effect on proliferation, differentiation, metabolism, and cell apoptosis. Excessive activation of this oncogenic pathway causes intensification of cell metabolism and proliferation and decreased apoptosis, and is often observed in patients with T-ALL [56].

CNAs in PTEN, PI3K, and AKT were noticed in primary T-ALL, which may suggest their role in the pathogenesis of leukaemia [74]. In a study conducted by Szarzyńska-Zawadzka et al., *PTEN* mutations and copy number variations in 162 paediatric patients with T-ALL diagnosis were analysed. Mutations and deletions were observed in 9 and 16% of patients, respectively. Coexistence of both deletion and mutation was observed in 8% of patients [72]. In a study conducted by Liu, *PTEN* mutation affected 14.0% of patients [41]. Similarly, Gutierrez et al. observed that *PTEN* deletions occurred in 8.7% of primary T-ALL patients. *PI3/AKT/mTOR* path activation may also occur as a result of *PI3K* or *AKT* mutation [74]. Lejman et al. observed CNAs in *PI3K/mTOR* pathway with a frequency of 23.26% in childhood T-ALL. Alterations in *PTEN*, *AKT1* (14q32.33), and *PIK3CD* (1p36.22) genes occurred in 11.63, 6.98, and 4.65%, respectively [42].

Patients with *PTEN* dysfunction were characterized by weaker response to prednisone treatment on day 8 after induction, and shorter 5-year ESF and higher risk of relapse compared to others with properly functioning *PTEN* genes [72]. Poorer outcome and response to treatment was observed only in the group with *PTEN* deletion [73]. The above correlation was not noticed in patients with only *PTEN* gene mutation. Moreover, the mutation of *AKT1* induced resistance to prednisone [56]. Another study confirmed that deletions in *PTEN* may contribute to treatment failure. However, this was not observed in the case of alterations in *PTEN-PI3K-AKT* [74].

MiR-19 has also been shown to inhibit the expression of *PTEN.* Additionally, the existence of an additional pathway of *PI3K/Akt* attenuation by *PTEN* using Ikaros/miR-26b axis was noted. It is worth mentioning that inactivation of *PTEN* increases the level of c-MYC expression by inhibiting its degradation by GS3Kβ [73].

The above-mentioned data indicate the need to conduct clinical trials involving *AKT*, *PI3K*, and *mTOR* inhibitors for targeted therapy.

#### 2.2.3. JAK-STAT Pathway

This pathway starts with the effect of IL-7 on its receptor, which leads to *JAK1* (1p31.3) and *JAK3* (19p13.11) phosphorylation. The next stage is the activation of STAT5 proteins, which regulate the transcription of genes responsible for antiapoptotic properties in the cell (BCL-2 BCL-XL MLC1) [1]. Therefore, constitutive activation of this pathway leads to uncontrolled proliferation of leukemic cells [56]. This path can be adjusted on several levels. The first one is activating IL-7R mutation, with a frequency of 6.8% [41]. The second is activating mutations in *JAK1*, *JAK3*, or *STAT5B* (17q21.2) [56]. *JAK3* mutations occur in 7.6% of T-ALL patients [41]. Activating mutations of *JAK1* gene were observed more often in T-ALL as compared to *JAK2* (9p24.1) in the BCP-ALL group. In patients with *JAK* gene mutation, more frequent co-mutations in the *IKZF1* gene and *CRLF2-* Xp22.3 and Yp11.3 (*CRFL2-r*) rearrangements were observed [56].

Another *JAK-STAT* aberrant modulator is *DNM2* inactivation, which leads to IL-7R overexpression on thymocytes [1]. In a study conducted by Liu et al., *DNM2* mutation occurred in 11% of T-ALL paediatric patients [41]. It has been observed that in T-ALL patients, *SH2B3* (12q24.12) mutations lead to aberrant activation of *JAK2/STAT* pathway [56]. It is worth mentioning that in 7% of patients with T-ALL diagnosis as a result of mutation and loss of *PTPN2* function, the *JAK-STAT* pathway was activated. A study carried out by Follini showed that aberrant activation of the *JAK-STAT* pathway is associated with induction of leukemic cell resistance to steroid therapy [1]. Alcantara et al. identified deletion in the protein tyrosine phosphatase nonreceptor type 2 (*PTPN2*—18p11.21) tumour suppressor gene. This deletion occurred in 6% of paediatric patients. In 54%, the deletion concerned both alleles, and in the remaining 46% of cases, it was monoallelic. An association was observed between the *PTPN2* loss and co-occurrence of αβ lineage and *TLX1* deregulation. Significantly, more patients with *PTPN2* deletion than *PTPN2* wild-type T-ALL had other accompanying disorders: *NUP214-ABL* gene fusion (21 vs. 7%), *IL7R/JAK-STAT* (74 vs. 41%), *NOTCH1/FBXW7* (85 vs. 63%), and *PHF6* (95 vs. 41%) mutations. Moreover, *PTPN2* deletion is an indicator of better outcome and glucocorticoid response. Lower 5-year OS and higher cumulative incidence of relapse (CIR) were observed in patients without *PTPN2* gene deletion compared to patients with a loss of *PTPN2* function: 78 vs. 92% and 26 vs. 8%, respectively [31].

A total of 178 paediatric patients undergoing AIEOP–BFM ALL 2009 protocol treatment were subjected to *CRLF2* (Xp22.3) gene expression level analysis; 14.6% of them had increased gene expression. *P2RY8/CRLF2* rearrangement was not observed in this group. In response to the question of whether increased *CRLF2* gene expression is of prognostic significance, minimal residual disease (MRD) and prednisone response were analysed. Patients with low expression responded to treatment, and prednisone resistance was observed in 35%. In contrast, glucocorticoid resistance was observed in 2 out of 3 patients with high *CRLF2* expression. Among patients classified as high-risk based on MRD measured on the 15th day after the induction of treatment, as much as 62% had *CRLF2* overexpression, compared to 23% of children with *CRLF2* downregulation [75].

#### 2.2.4. RAS Pathway

In the Ras signal, we distinguish three types of genes involved: *NRAS* (neuroblastoma Ras; 1p13.2), *KRAS* (Kirsten Ras; 12p12.1), and *HRAS* (Harvey Ras; 11p15.5). Interestingly, studies carried out on mouse models proved that co-occurrence of mutations of these genes with *NOTCH1* and *IL-7R* mutations and inactivation of *EZH2* may be responsible for leukaemia induction. ETP-ALL cases were characterized by more frequent occurrences of *N-/K-Ras* mutation activation. Additionally, this disorder was associated with *TLX1/TLX3+* and *HOXA+* patients [76]. *N-Ras* gene mutation was observed with a frequency of 6.2% [57], 7.6% [41], 10.8% [17], and 7% [77] among patients with T-ALL. The frequency of *K-Ras* mutation was 2.1% [57], 5.9% [17], and 11.4% [77]. These genes were more frequently mutated in patients with *CDKN2B* hypermethylation [17]. Additionally, Zhang and Richter-Pechańska suggested that it may be associated with relapse and glucocorticosteroid resistance [11,78]. Iacobucci et al. observed that Ras pathway mutations appeared during treatment and were dominant in secondary disease, and are related to high risk and poor outcome [9].

### 2.3. Cell Cycle Regulators

*CDKN2A/2B* (9p21.3) are among the most frequent CNAs occurring in paediatric patients with T-ALL (Table 3). Those genes, which belong to the INK family and lie on chromosome 9p21.3, demonstrate a tumour suppressive effect. *CDKN2A/2B* gene inactivation can proceed through deletion, mutation, or epigenetic silencing by hypermethylation of the promoter. The loss of function of p15 and p16 proteins encoded by these genes leads to uncontrolled proliferation of neoplastic cells [79].

*CDKN2A/2B* deletion is more common in T-ALL in comparison to BCP-ALL [79]. *CDKN2A* deletion was observed in 66% of paediatric patients with T-ALL and *CDKN2B* in 55% [11]. Moreover, this deletion occurred at higher frequency in patients with cortical T-ALL (47%) than in those with ETP-ALL (3%) [80]. It is important to mention that biallelic deletion was associated with all T-ALL cases compared to BCP-ALL cases, in which monoallelic deletion dominated. Hypermethylation is one way for inactivation to be observed more often in the *CDKN2A* gene [79]. In contrast, Jang observed that the main method of gene inactivation was deletion for *CDKN2A*, and both deletion and hypermethylation for *CDKN2B* [17].

The prognostic value of *CDKN2A/2B* gene inactivation for progression of the disease and response to treatment is highly debatable, due to the small number of patients and the heterogeneous nature of T-ALL. Interestingly, patients with *CDKN2A/2B* gene deletion were characterized by older age at diagnosis and a higher number of white blood cells (WBCs) in peripheral blood. These features are factors in poor ALL outcomes. It is also worth noting that deletion was correlated with lower event-free survival (ESF) versus lack of deletion, at 12.5 and 87.5%, respectively. T-ALL patients with *CDKN2A/2B* deletion had a significantly lower survival rate than patients without deletion [79]. As opposed to Agarwal, Genesca et al. reported that the prognosis for patients with *CDKN2A/2B* inactivation was better. These T-ALL patients did not require intensification of therapy or marrow transplant. Better overall survival (OS) rates over 3 years were reported in patients with *CDKN2B* deletion. This correlation was not observed in patients with *CDKN2A/ARF* deletion [80].

In a study performed on 102 patients with T-ALL, Jang et al. observed the above-mentioned gene deletions in 34.3%. Similarly, to Agarwal, they found that the biallelic deletion was more often in the *CDKN2A* gene than in the *CDKN2B* gene, at 95.3 and 76.3%, respectively. They also noted that methylation of both *CDKN2A/2B* genes is more common in patients without deletion of these genes. Deactivation of *CDKN2B* gene was mainly caused by epigenetic silencing by hypermethylation of promoter. Additionally, it is worth noting that *CDKN2B* deletion was related to younger age at diagnosis, lower white blood cell (WBC) count and increased blast percentage, and more mature phenotype. In contrast, *CDKN2B* hypermethylation was observed in practically all ETP-ALL cases. Children with biallelic deletion or >45% hypermethylation were compared with patients with monoallelic deletion and hypermethylation at levels below 45%. The second group was characterized by better prognosis, longer EFS, and 3-year OS compared to the first group (59.1 vs. 35.9%, 85.2 vs. 43.0%, respectively) [17].

Inactivation of the cyclin-dependent kinase inhibitor 1B gene, located on the chromosome 12 *CDKN1B* (12p13.1), is response for uncontrolled cell proliferation and has been observed in leukaemias [81]. In research conducted by Colomer-Lahiguer, deletion of this gene was found in about 12% of paediatric T-ALL patients. Patients with *CDKN1B* deletion were characterized by high *HOXA* subtype and less frequent coexistence of CNAs in *CDKN2A*/2B genes. Moreover, the co-occurrence of *CDKN1B* deletion and *MEF2C* dysregulation was noted. Genetic changes involving *MEF2C* were found in 54% of cases with *CDKN1B* dysregulation, in comparison to 14% in patients with wild-type *CDKN1B*. Additionally, a link between poor response to steroids and *MEF2C* dysregulation has been demonstrated [13].

Cycline D2 belongs to the group of proteins involved in the regulation of the cell cycle and is encoded by the *CCND2* gene (12p13.32), located on chromosome 12, whose changes have been observed in numerous cancers [82]. A study by Clappier et al. compared the expression of *CCND2* in a population of healthy thymus cells and cells of patients with T-ALL. It was observed that healthy cells had low *CCND2* expression compared to cells in 3 translocated patients out of 89 T-ALL cases, characterized by massive overexpression of this gene. This discovery led to the suspicion that *CCND2* overexpression in T-ALL cells has a role in T-ALL development [83].

### 2.4. Kinase Signalling

In recent years, the efficacy of tyrosine kinase inhibitors (TKIs) has been observed in patients with platelet derived growth factor b (*PDGFRb*) activation [84] (Table 4). As a result of this discovery, a new group of patients was described who showed gene expression similar to Ph-like ALL, but had no fusion between *BCR* and *ABL1*. Among this group, cases with *PDGFRB* (5q32) rearrangements were observed. *EBF1-PDGFRB* (5q33.3), which determined good response to ABL1 TKI, was described. Heilman et al. reported three consecutive partner genes, *SATB1* (3p24.3), *AGGF1* (5q13.3), and *DOCK2* (5q35.1), which had *PDGFRB* rearrangements in T- and BCP-ALL patients [85]. Bielorai was the first to describe a paediatric T-ALL case of a patient with *PDGFRB*-R and t(5;14) (q33;q32) who responded well to imatinib treatment [86]. In contrast, Zhang observed a *PDGFRBC843G* mutation in a Ph-like ALL patient, which conditioned resistance to ABL TKIs and sensitivity to the CHZ868 multitarget kinase inhibitor [87].

*ABL1* (9q34.12) fusion proteins were observed in 8% of T-ALL cases and were linked to leukemic survival and proliferation of cells, and provided sensitivity to tyrosine kinase inhibitors. Among all partner genes, *NUP214* (9q34.13) is the most common and characteristic, and occurs in 6% of childhood T-ALL cases. Other genes, such as *BCR* (22q11.23), *ETV6 (12p13.2)*, *ZBTB16* (11q23.2), and *EML1* (14q32.2), are found more frequently in other cancers and are rarely observed in T-ALL [88]. Amplification of the *NUP214-ABL1* gene was observed in 5–6% and was linked to therapy failure and relapse [42]. *BCR-ABL1* fusion due to t(9;22)(q34;q11.2) was observed in 9% Ph+ALL patients, but taking into consideration the whole ALL population, it was observed in only 0.3%. Rearrangement between 9q34 and 12p13 was involved in *ETV6-ABL1* fusion [88]. Chen et al. observed *ZBTB16-ABL1* fusion in two cases. This rearrangement co-occurred with other genetic alterations, such as *NOTCH1*, *PTEN*, *MYCN*, and *PIK3CD* and *ZEB2* mutations. Both cases were characterized by a very aggressive process. In an in vitro study, the activity of TKI on cells with *ZBTB16*-*ABL1* and *BCR*-*ABL1* was checked, showing similar sensitivity to this therapy [89]. The extremely rare phenomenon of translocation (9;12)(q34;p13) leads to fusion between the *EML1* and *ABL1* genes. Hagemeijer observed *EML1*-*ABL1* in only one patient with T-ALL [88]. The assessment of *ABL1* rearrangements is extremely important in the context of the possibility and reasonableness of using targeted therapy with TKI.

The *FLT3* (13q12.2) gene regulates haematopoiesis and is responsible for the formation of tyrosine receptor class III kinase. The tyrosine kinase activation occurs as a result of internal duplication of the *FLT3/ITD* tandem, which is most frequently observed in T-ALL [18]. Mutations of this gene were observed in 3% of patients with T-ALL [47]. *FLT3* overexpression was observed more commonly in ETP-ALL cases [41]. Interestingly, this mutation was more common in BCP-ALL compared to T-ALL (7 vs. 3.8%) [77]. Mutation of the *FLT3* gene in acute myeloblastic leukaemia affects every third patient and has a proven negative prognosis [47]. In T-ALL, due to the rare occurrence of this gene aberration and the small size of the study group, no effect of *FLT3* mutation on patients’ prognosis was discovered.

### 2.5. T-ALL Rearrangements

*SIL-TAL1* (1p32; 1p33) gene rearrangements in paediatric patients with T-ALL are observed with a 16–26% frequency [90] (Table 5). In a study carried out on a group of 68 patients (26 paediatric), *SIL-TAL1* gene fusion was observed in 38.5% of children. The group of patients with *SIL-TAL1* gene fusion was characterized by younger age, higher WBC count at the time of diagnosis, a higher level of lactate dehydrogenase (LDH), and increased frequency of disseminated intravascular coagulation (DIC) and acute tumour lysis complications [90,91]. Therefore, the occurrence of this fusion was associated with worse prognosis. A study in a group of 101 children with T-ALL did not show any differences in minimal residual disease (MRD), 5-year EFS or relapse-free survival (RFS) between patients with and without the fusion. The possibility of worse response to remission induction therapy was observed because higher MRD was observed more often among patients with *SIL-TAL1* gene rearrangements [92]. Additionally, in a study conducted by Ohki, a higher incidence of *SIL-TAL1* fusion was observed in patients in the EGIL-IV and -III groups, 55 and 30%, respectively. Interestingly, patients with and without fusion did not show any differences. However, in group IV, patients with *SIL-TALI1* were characterized by higher ESF (90%) in comparison to others without fusion (57.1%) [8]. *TAL1* gene deletion is a common abnormality, observed with a frequency of 25% of patients with T-ALL [65]. Wang et al. achieved similar results. *SIL-TAL1* deletion occurred in every fifth patient with T-ALL [66]. Both Chen and Wang suggested that *TAL-1* gene deletion detection may be useful in assessing MRD in T-ALL patients [65,66]. In another study, deletion of TAL1 was observed in 3 out of 39 patients under 18 years of age and was associated with a higher WBC count [67].

Acute leukaemia with a *PICALM-MLLT10* fusion gene (originally called *CALM-AF10*) is generated by t(10;11)(p12-13;q14-21) translocation, and has been very rarely reported in patients with T-ALL, at an overall rate of 10%, including both adults and children [93]. The presence of *PICALM-MLLT10* has been associated with a poor prognosis, and several studies included very few children with T-ALL [94]. Literature on the *PICALM-MLLT10* issue is quite limited, and no definitive conclusions have been drawn on the incidence and prognostic impact of this fusion transcript among children with T-ALL. Nigro et al. reported that *PICALM-MLLT10* fusion transcript occurs in 7% of children with T-lineage ALL and is not associated with poorer outcomes for patients treated with contemporary intensive chemotherapy [93]. This issue requires a multinational study to enable a meaningful retrospective analysis.

*SET-NUP214* (9q34.11) was identified as a novel recurrent fusion gene in T cell leukaemia by Van Vlierberghe at al. They showed that *SET-NUP214* may contribute to T-ALL pathogenesis by inhibition of T cell maturation through the transcriptional activation of *HOXA* genes, often predicting a poor outcome for patients [95]. The fusion transcript may be regarded as a potential minimal residual disease marker for *SET-NUP214*-positive patients [96]. Interstitial deletion, which results in *SET/NUP214* fusion, has been described in 6 of 40 children with del 9q34 region in a study conducted by Papenhausen at al. [97].

### 2.6. Epigenetic Regulators

The polycomb protein complex (PRC2) includes *EZH2* (7q36.1), *EED* (11q14.2) or *SUZ12* (17q11.2). Mutations inactivating these genes lead to the acquisition of cell resistance to mitochondrial apoptosis and the drugs for which this mechanism is the grip point. In the Ariës study, mutations in the *PRC2* complex affected every five childhood T-ALL cases [98] (Table 6). Another study showed a lower incidence; mutations in the *EZH2* and *EED* genes were found in 3.3% of patients [99]. High expression of *EZH2*, *EED*, and *SUZ12* genes was observed in 75, 60, and 58.3% of patients in whom these genes were expressed. The remaining cases were characterized by low expression [100]. In addition, Schafer et al. observed that paediatric patients with T-ALL were characterized by a significantly higher incidence of hypermethylation of the *EZH2* gene promoter compared to healthy cells [99]. It has been shown that patients with high expression of *EZH2* and *EED* genes are less likely to achieve disease-free survival (DFS) [100]. On the other hand, the *PRC2* mutation was closely related to poorer response to drug treatment, but no correlation between inactivation of the *PRC2* complex and shorter OS was reported [98].

### 2.7. Tumour Suppressors

*PHF6* (Xq26.2), located on the X chromosome, acts as a suppressor gene, and its deletion may be relevant in T-ALL development [42] (Table 7). The literature reports *PHF6* mutation occurring in 14, 26.7, 16, and 5.4% of patients with paediatric T-ALL [42,91,101,102]. In addition, *PHF6* mutation is more prevalent in men (32.0%) than women (2.5%) [102]. In contrast, Wang did not observe differences in the occurrence of *PHF6* alterations between genders. In his study, the frequency of *PHF6* alterations in children with T-ALL was 5.4% for mutations and 2.5% for deletions [91]. Alterations in the *PHF6* gene were much more frequently observed in adult patients with T-ALL compared to children (38 vs. 16%) [102]. A similar trend was observed by Wang (18.6 and 5.4%, respectively) [91]. On the other hand, CNAs in *PHF6* were not reported in cases with BCP-ALL [102]. The molecular genetic markers most frequently associated with *PHF6* mutations were *NOTCH1* mutations, *SET-NUP214* rearrangements, and *JAK1* mutations. It has been suggested that the *PHF6* gene may be relevant in inducing resistance to steroids [42,103]. Wang compared patients with and without *PHF6* mutations and did not observe any differences between OS and DFS [91].

The role of the *TP53* gene as a tumour suppressor in the pathogenesis of many cancers is well known. Under normal conditions, the level of *TP53* (17p13.1) is low because it occurs mainly in inactive form associated with *MDM2.* However, when DNA is damaged, it is activated for repair. The levels of *MDM2* and *RB* are regulated by the *CDKN2A* gene, therefore deactivation of *CDKN2A*, which is common in T-ALL patients, leads to *TP53* and *RB* pathway disturbances [56]. *RB* alterations were observed in 9.5% of T-ALL patients [41]. Deregulation of *TP53* also occurs through a mutation, whose involvement in T-ALL recurrence was shown by Richter-Pechańska et al. [11]. Additionally, the presence of the mutation was associated with a reduced response to treatment and shorter OS. The pathway of *TP53* is also affected by hypermethylation of promoters and miRNA-126 and -181a, involved in gene expression [56].

Deletion of exons 13-17 in the *RB1* gene (13q14.2) is frequently observed in various types of tumours, including retinoblastoma, breast cancer, and osteosarcoma, and the presence of a potential hotspot for recombination in the region was predicted [104]. CNAs in *RB1*, which have a negative impact on the cell cycle, were reported in 24.4% of T-ALL patients [42]. In contrast, much rarer occurrences were observed in a study conducted on 264 children and young adults, in which the frequency of *RB1* mutations was determined to be 9.5% [41]. Deletion containing the *RB1* gene was reported in 2 of 36 childhood T cell ALL cases (5.6%) by Olsson and in 8.3% (*n* = 2) by Krzanowski [49,64].

### 2.8. Translation and RNA Stability

Ribosomes are responsible for protein synthesis, which is necessary for the proliferation of leukemic cells. In paediatric patients diagnosed with T-ALL, anomalies were observed in genes encoding cytoplasmic ribosomal proteins L5, L10, L11, and L22, which are components of subunit 60S (Table 8). Every fifth child with T-ALL had mutations and deletions of *RPL10* (Xq28), *RPL5* (UL18; 1p22.1), and *RPL22* (1p36.31) [105]. The most frequent mutation in paediatric patients, with a frequency of 6–8.2%, was observed in gene encoding cytoplasmic ribosomal protein L10. Interestingly, it usually involved missense mutations at residue R98 (Arg98Ser) [106,107]. Mutation in *RPL5* affects 1.6% of paediatric T-ALL patients. Leukemic cells with the current *RPL10R98S* mutation are characterized by overexpression of BCL2 protein, which prevents apoptosis. This discovery may allow the use of BCL-2 targeted therapies [107].

## 3. Genetic Aberrations Involved in Relapse

A study conducted by Richter-Pechanska on a group of 214 T-ALL patients showed several genes that may be responsible for disease relapse and are much more common in relapsed patients than original patients [11].

In a study conducted by Kunz et al., *NT5C2* mutation was noted in two patients with primary disease, while it was observed in 38.5% *(n* = 5) of relapsed patients [108]. Similar to the above-mentioned study, the most frequent mutation detected in patients with relapse was *NT5C2* gene activation mutation, which was observed in 24%. However, it was suggested that this does not initiate relapse, as this mutation was observed in 71% of patients before relapse [11]. Additionally, it has been shown that this mutation is related to cell resistance to chemotherapy (6-mercaptopurine and 6-thioguanine) [78,108].

The second occurrence, concerning 13.4% of patients with relapse, was *TP53* aberration. This disorder was much less common in primary disease (1.36%). It is worth mentioning that deletions or single nucleotide variants (SNVs) are often accompanied by other CNAs. Additionally, the coexistence of gene mutations supervising DNA integrity was observed, with *TP53* and *USP7* (16p13.2), and *MSH6* (2p16.3), as factors of relapse and high mortality [11]. *USP7* mutations were noticed in 11.7% of patients with T-ALL diagnosis [41].

Another three mutations showed a connection to relapse. Mutation of *RAS* genes (*KRAS* and *NRAS*) was observed in eight patients with T-ALL relapse. It was interesting that as many as six of them died within 3 months after the relapse [11].

Mutation of the *CNOT3* (19q13.42) gene with tumour suppressor function occurs with a frequency of 8% in adult patients with T-ALL. In Richter-Pechanska’s study, all patients in whom *CNOT3* mutation was observed died due to relapse. The same observation was noted for IL-7 receptor mutation [11].

Mutations and gene deletions were also observed in paediatric patients whose protein products were involved in changes in chromatin conformation: *PHF6* and *EZH2.* In a study conducted by Liu in 2017, *PHF6* was observed in 18.9% of paediatric patients with T-ALL. It is also worth mentioning a mutation of ribosomal protein gene involved in the *RPL10* translation process, which was observed in 6.1% of children with T-ALL [41].

## 4. Chromothripsis

Chromothripsis, as an expression of chromosomal instability, leads to complex reconfigurations in a short time [109]. Multiple CNAs occur during a single event, and this can result in the loss of chromosome fragments encoding suppressor genes and the formation of fusion genes that are relevant for oncogenesis. Therefore, its occurrence is observed in many diseases and cancers, but not often in T-ALL [110]. Ratnaparkhe et al. showed that the genomic landscape of patients with ataxia telangiectasia and T-ALL differs from that of sporadic ALL with a much higher frequency of chromothriptic events. They detected a high frequency of chromothriptic events in these tumours, specifically on acrocentric chromosomes [111]. Lejman et al. described the case of a child with T-ALL with chromothripsis including chromosome 11. The scientists suggested that this process is linked to worse prognosis. Further research is needed in this area [42].

## 5. Conclusions

The last decade and the development of innovative technologies such as next-generation sequencing (NGS) have made it possible to accurately characterize the genetic changes associated with leukaemia transformation. However, T-ALL in children remains a major challenge due to its heterogeneous course, rare occurrence, and high heterogeneity. The search for predictive genetic markers in T-ALL is ongoing, and will provide critical and relevant information not only on the classification of patients to the appropriate risk and treatment groups, but also on markers for the evaluation of minimal residual disease, risk of relapse, resistance to given chemotherapies, and as grip points for targeted therapies.

The characterization of molecular alterations with prognostic impact in T-ALL may be very helpful for early identification of patients at high risk of failure, for whom more intensive treatments, including allogeneic hematopoietic stem cell transplantation, may be considered.

## Figures and Tables

**Table 1:** Transcription factors in T-ALL (T-cell acute lymphoblastic leukaemia).

Gene	Locus	Alterations	Partners gene	Incidence	Relevance	References
*TAL1* *(SCL, TCL5)*	1p32	Overexpression with coexisting *LMO1/2*	*STIL* (1p33)	30–37.5%	Adverse, potential MRD marker	[29,33]
		deletions	-	7.7–25%		[65,66,67]
*TLX3 (HOX11L2)*	5q35	Overexpression	*BCL11B* (14q32)	20–24%	Adverse	[29]
*TLX1 (HOX11)*	10q24		*TCRD* (14q11)	3–8%	Favorable	[29,34]
		Expression level	*TCRB* (7q35)	OE ^1^ 19.7%, LE ^2^ 28.9%	N/A ^3^	
*NKX2-1* *NKX2-2*	14q13 20p11	Overexpression	-	5.9%	Unidentified	[29]
*LMO1* *(TTG1)*	11p15	Translocations	*TRB* (7q34) *TRA* (14q11.2) *TRD* (14q11)	<1%	Unidentified	[41]
*LMO2* *(TTG2)*	11p13	Translocations	*TRA* (14q11.2) *FOXJ3* (1p34)	7.7% 5%	Unidentified	[41]
		Deletions	del(1)(p12p13)			
*MEF2C*	5q14.3	Overexpression	*CLINT1* (5q33.3)	2.5%	Adverse, reduce response to steroids	[13,29]
*BCL11B* *(CTIP2)*	14q32.2	Mutations	-	2–9%	Unidentified	[47,48]
*KMT2A* *(MLL)*	11q23	Rearrangements	Various partners gene	4–12%	Adverse	[35,36,37,38,39,40]
*MYB*	6q23	Rearrangements	*TCRB* (7q34) *SLC12A9* (7q22) *PLAGL1* (6q24) BDP1 (95q13) *CHMP1A* (16q24)	4.17%	Unidentified	[41]
		Mutations	-	4.92%		[41]
		Amplifications	-	12.5–12.94%		[41,42]
*MYC*	8q24	Translocations	*TRA* (14q11)	6.1%	Unidentified	[44]
*RUNX1* *(AML1, CBFA2, PEBP2AB)*	21q22.12	Mutations	-	12.7%;	Adverse	[17]
*ETV6* *(TEL)*	12q13.2	Deletions	-	5.6–6%	Unidentified	[49,50]
		Translocations	*ABL1* (9q34), *CTNNB1* (3p22.1)	<1%		[41]
*GATA3*	10p14	Inactivating mutations	-	9% of paediatric patients with ETP-ALL	Adverse	[52,53]
*LEF1* *(TCF10)*	4q25	Deletions	-	7–24.5%,	Divided	[41,43,55,56,57]
*WT1*	11p13	Mutations	-	9.1–13.2%	N/A	[41,58]
*NF1*	17q11.2	Deletions	-	7.4%	adverse, resistance to treatment induction	[43]
*IKZF1* *(LYF1)*	7p12.2	Loss of its function	-	7.4%	Adverse, especially in B cell precursor (BCP)-ALL	[41,43,49,63]
		Deletions		2.8%		
		Mutations		2%		
*PAX5* *(BSAP)*	9p13.2	Loss of its function, deletions and mutations	-	11–14%	Unidentified	[42,43,49]
*IKZF2*	2q34	loss of its function, deletions and mutations	-	3.4%	Unidentified	[63]

^1^ OE—overexpression, ^2^ LE—low expression, ^3^ N/A—no association.

**Table 2:** Signalling pathway regulators in T-ALL.

Gene	Locus	Alterations	Incidence	Relevance	References
*NOTCH1*	9q34.3	Activating mutations	51.1–74.6%	Favorable, targeted therapy—γ-secretase inhibitors (GSI)	[41,68]
		t(7;9)(q34:q34.3)	<1%		
*FBXW7*	4q31.3	Inactivating mutations	22.4–23.9%	Unidentified	[41,68]
*PIK3CD* *(PI3K-DELTA)*	1p36.22	Deletions	4.65%	Unidentified, targeted therapy—PI3K inhibitors	[42]
		Mutations	1.9%		[41]
*AKT*	*14q32.33*	Deletions	6.98%	Adverse, resistance to steroids, targeted therapy—AKT inhibitors	[42,56]
*PTEN*	10q23.31	Mutations	9–14%	Adverse, reduced response to treatment	[41,42,72,73,74]
		Deletions	8.7–16%		
*DNM2*	19p13.2	Inactivating mutations	11%	Unidentified	[41]
*IL7R*	5p13	Activating mutations	6.8%	Unidentified	[41]
*JAK3*	19p13.11	Activating mutations	7.6%	Unidentified	[41]
*PTPN2*	18p11.21	Deletions	6%	Favorable, good steroid response	[31]
*CRLF2*	Xp22.3 and Yp11.3	Overexpression	14.6%	Adverse, resistance to steroids	[75]
*N-RAS*	1p13.2	Activating mutations	6.2–10.8%	Adverse, resistance to steroids, associated with relapse	[9,11,17,41,57,77,78]
*K-RAS*	12p12.1	Activating mutations	2.1–11.4%	Adverse, resistance to steroids, associated with relapse	[9,11,17,57,77,78]

**Table 3:** Cell cycle regulators in T-ALL.

Gene	Locus	Alterations	Incidence	Relevance	References
*CDKN2A* (p16(INK4A))	9p21.3	Deletions, mutations, promoter hypermethylation	66%	Divided	[11,17,79,80]
*CDKN2B* (p15(INK4B))	9p21.3	Deletions, mutations, promoter hypermethylation	55%	Divided	[11,17,79,80]
*CDKN1B* (p27(KIP1))	12p13.1	Deletions; cooccurrence with *MEF2C* dysregulation	12%	Adverse, reduced response to steroids	[13]
*CCND2*	12p13.32	Overexpression	3.4%	Unidentified	[83]

**Table 4:** Kinase signalling in T-ALL.

Gene	Locus	Alterations	Partner Genes	Incidence	Relevance	References
*PDGFRb*	5q32	Rearrangements	*EBF1* (5q33.3), *SATB1* (3p24.3), *AGGF1* (5q13.3) *DOCK2* (5q35.1)	single cases	Adverse, tyrosine kinase inhibitor (TKI) treatment	[85,86]
*ABL1*	9q34.12	Rearrangements, amplifications	*NUP214* (9q34.13)	5–6%	Adverse, TKI treatment	[42,88,89]
			*BCR* (22q11.23), *ETV6 (12p13.2)*, *ZBTB16* (11q23.2), *EML1* (14q32.2)	<1%		
*FLT3* *(STK1)*	13q12.2	Mutations	-	3.0–3.8%	N/A	[47,77]

**Table 5:** T-ALL rearrangements.

Fusion	Locus	Alterations	Incidence	Relevance	References
*SIL-TAL1*	1p32; 1p33	Rearrangements	16–38.5%	Adverse	[8,90,91,92]
*PICALM-MLLT10* (*CALM-AF10)*	11q14.2; 10p12.31	Rearrangements, t(10;11)(p12-13;q14-21)	7%	Adverse/unidentified	[93,94]
*SET-NUP214*	9q34.11; 9q34.13	Rearrangements	15%	Unidentified; potential minimal residual disease (MRD) marker	[96,97]

**Table 6:** Epigenetic regulators in T-ALL.

Gene	Locus	Alterations	Incidence	Relevance	References
*EZH2* *(KMT6A, ENX1)*	7q36.1	Inactivating mutations and promoter hypermethylation	3.3% (adult)	Adverse, less opportunity to obtain disease-free survival (DFS)	[100]
*EED*	11q14.2	Inactivating mutations			
*SUZ12* (JJAZ1)	17q11.2	Inactivating mutations	0–7.4%	Adverse	[43,57]

**Table 7:** Tumour suppressors in T-ALL.

Gene	Locus	Alterations	Incidence	Relevance	References
*PHF6*	Xq26.2	Mutations	5.4–26.7%	Adverse, resistance to steroids	[41,42,91,101,102,103]
		Deletions	2.5%		[91]
*TP53*	17p13.1	Mutations and promoter hypermethylation	5%	Adverse, reduced response to treatment and shorter OS	[56]
*RB1*	13q14.2	Mutations	9.5%	-	[41]
		Deletions	5.6–8.3%		[49,64]

**Table 8:** Translation and RNA stability in T-ALL.

Gene	Locus	Alterations	Incidence	Relevance	References
*RPL5*	1p22	Inactivating mutations	1.6%	Unidentified	[106]
*RPL10*	Xq28	Inactivating mutations	6–8.2%	Unidentified; BCL-2 targeted therapies	[105,106,107]
*RPL22*	1p36.31	Inactivating mutations	<1%	unidentified	[106]

## Abbreviations

AKT1	the serine-threonine protein kinase
ALL	acute lymphoblastic leukemia
BCP-ALL	B-cell precursor acute lymphoblastic leukemia
BSAP	B-cell-specific protein activator factor
CIR	cumulative incidence of relapse
CNAs	copy number alterations
DFR	disease free of recurrence
DIC	disseminated intravascular coagulation
DR	Downregulation
EGIL	The European Group for the Immunologic Classification
ESF	event-free survival
ETP-ALL	Early T-cell precursor ALL
GSI	γ-secretase inhibitors
HD	heterodimerization domain
JAK 1	janus kinase 1
LBL	acute lymphoblastic lymphoma
LDH	lactate dehydrogenase
MRD	minimal residual disease
NGS	new generation sequencing
OE	Overexpression
OS	overall survival
PDGFRb	platelet dderived growth factor b
PEST	(-Pro-Gln-Ser-Thr-) domain.
PIP2	phosphatidylinositol (4,5) P2
PIP3	phosphatidylinositol (3,4,5) P3
PRC	polycomb group complex
RFS	relapse-free survival
SNV	single nucleotide variants
STAT5B	signal transducer and activator of transcription 5B
T-ALL	T-cell acute lymphoblastic leukemia
TCR	T cell antigen receptor
TKI	tyrosine kinase inhibitor
WBC	white blood cell
